# Hemi-root replacement with diagonal resection of the aortic root preserving the ostium of the left coronary artery and sacrificing the right coronary artery for an infected pseudoaneurysm: a case report

**DOI:** 10.1093/jscr/rjac129

**Published:** 2022-04-04

**Authors:** Hideki Isa, Tomonori Shirasaka, Shingo Kunioka, Hiroyuki Kamiya

**Affiliations:** Department of Cardiac Surgery, Asahikawa Medical University, Asahikawa, Japan; Department of Cardiac Surgery, Asahikawa Medical University, Asahikawa, Japan; Department of Cardiac Surgery, Asahikawa Medical University, Asahikawa, Japan; Department of Cardiac Surgery, Asahikawa Medical University, Asahikawa, Japan

## Abstract

Either the Bentall-De Bono operation or the valve-sparing aortic root replacement is commonly chosen for aortic root management. However, if the preoperative condition is poor, a simpler technique is preferred; therefore, we performed hemi-root replacement with diagonal resection of the aortic root preserving the left coronary sinus of Valsalva. Because reimplantation of the left coronary artery is not required, this technique may shorten operative time and reduce coronary malperfusion, a condition characterized by reduced transit flow time and reduced cardiac contractility.

## INTRODUCTION

The standard treatment for aortic root lesions is the Bentall-De Bono operation or the valve-sparing aortic root replacement [1]. However, if the patient’s preoperative condition is poor, a simpler technique is preferred; therefore, for an aortic root-infected pseudoaneurysm, we performed hemi-root replacement with diagonal resection of the aortic root preserving the left coronary sinus of Valsalva. Because reimplantation of the left coronary artery (LCA) is not required, this technique may shorten operative time and reduce coronary malperfusion, a condition characterized by reduced transit flow time and reduced cardiac contractility. This technique also may be an option in some cases of acute aortic dissection accompanied by an impaired aortic valve and dissection of the right and non-coronary sinuses of Valsalva.

## CASE REPORT

**Figure 1:**
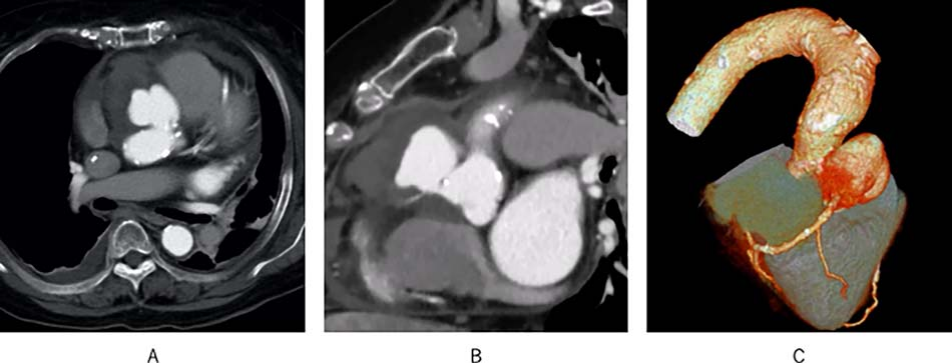
Preoperative contrast-enhanced CT; (**A**) a pseudoaneurysm was found in the aortic root; (**B**) the RCA appeared to originate from the pseudoaneurysm; (**C**) 3D CT showed the same results.

A 72-year-old woman with an elevated inflammatory response (white blood cell: 15 030/μl, C-reactive protein: 7.74 mg/dl) was transferred to our department for surgery of an aortic root pseudoaneurysm. Transthoracic echocardiography (TTE) revealed severe tricuspid regurgitation, moderate aortic stenosis (AS) and an aneurysm with a large ostium near the origin of the right coronary artery (RCA). Moreover, contrast-enhanced computed tomography (CT) revealed the RCA appeared to arise from the aneurysm (Fig. 1). The patient was scheduled to undergo elective surgery. However, on the third day of admission, creatine kinase MB increased from 2.6 to 23 U/l, urine volume decreased to about 10 ml/hour and 6 l of oxygen was required, so emergency surgery was performed.

In the operating room, the left internal thoracic artery and saphenous vein graft (SVG) were harvested first; however, the patient’s SpO_2_ dropped to 82%, and cardiopulmonary bypass (CPB) was established via the right femoral approach because of strong adhesions in the pericardium. Antegrade and retrograde cold blood cardioplegia were administered intermittently.

A pseudoaneurysm between the aortic root and the right ventricle was detected. The pseudoaneurysm perforated into the wall of the right ventricle, causing dissection of the ventricular wall. A 1.5-cm diameter orifice of the pseudoaneurysm was found just above the RCA orifice, and an encapsulated abscess was found on the basal side of the pseudoaneurysm wall (*Escherichia coli* was detected in the aneurysm culture). The patient was diagnosed with an infected aortic root pseudoaneurysm.

**Figure 2:**
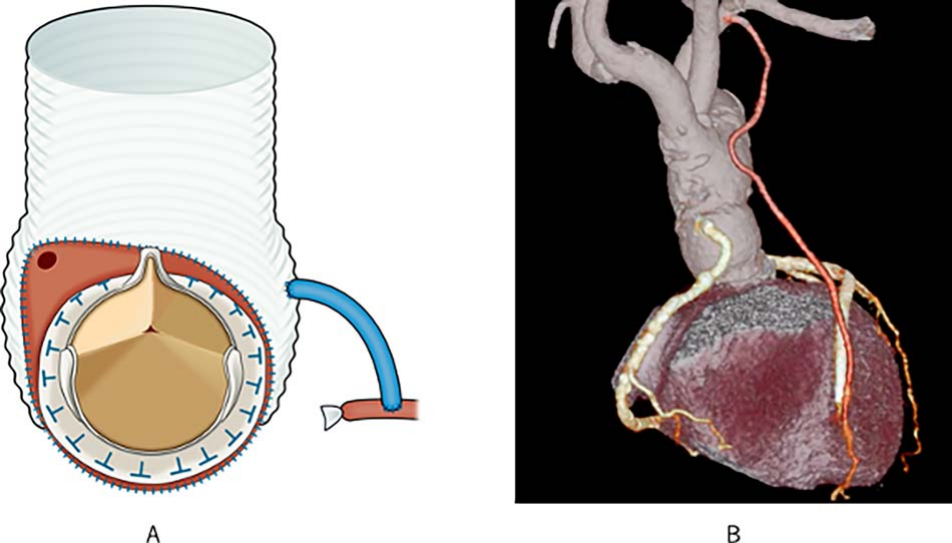
Post-operative schema and 3D CT; (**A**) the aortic root was resected diagonally, and AVR was performed; next, the vascular graft was anastomosed to the aortic root; finally, the right coronary artery was translocated using an SVG; (**B**) post-operative 3D CT showed that the pseudoaneurysm disappeared, and all grafts were patent.

We considered performing the Bentall-De Bono operation; however, due to preoperative circulatory deterioration, we decided to perform hemi-root replacement while preserving the left sinus of Valsalva and the ostium of the LCA, resection of the right and non-coronary sinus of Valsalva, translocation of the RCA with vein graft and aortic valve replacement (AVR), as shown in Fig. 2 and Supplementary Video 1.

First, the aortic root was diagonally resected with sufficient debridement of the infected tissues. Thereafter, AVR was performed, and a 26-mm J graft 1 branched (Japan Lifeline Inc, Tokyo, Japan) was anastomosed to the aortic root. Finally, the RCA was translocated using an SVG.

The patient could not be weaned from the CPB, and veno-arterial extracorporeal membrane oxygenation (VA-ECMO) was introduced instead. The operation was completed with an open chest. CPB time was 343 minutes and aortic cross clamp time was 223 minutes. VA-ECMO was halted on the fifth post-operative day and the patient recovered gradually. Post-opeative TTE shows improvement in the ejection fraction and there were no complications with the movement of the aortic valve. However, the patient died 4 months after the operation; the cause of death was determined at autopsy to be septicemia secondary to pneumonia.

## DISCUSSION

In consideration for the patient’s preoperative condition, we performed hemi-root replacement with AVR as an alternative to the Bentall-De Bono operation, which required reimplantation of the LCA. The Bentall operation is the gold standard for aortic root lesions. However, because of the lower durability of a bioprosthetic valve and the necessity of anticoagulation medication to prevent thromboembolism when using a mechanical valve, the valve-sparing aortic root replacements, such as the remodeling operation by Sarasm and Yacoub and the reimplantation operation by David and Feindel, have also been performed [2, 3]. However, the entire sinus of Valsalva is replaced and the bilateral coronary arteries are reimplanted in the operations described above, thus requiring the replacement of tissue that retains its normal structure. Shrestha *et al*. stated that although aortic root reimplantation was effective, it was not superior to the Bentall operation in older patients. They attributed this to the prolonged operative time and aortic interruption time due to technical difficulty [4]. In this case, since the preoperative condition was poor, it was necessary to complete the surgery through a simple and minimally invasive procedure.

In our case, the right coronary and the non-coronary sinus of Valsalva had to be replaced. In addition, the patient had moderate AS preoperatively, which required AVR. Though partial aortic root remodeling with AVR and translocation of the RCA were theoretically possible, we judged that the suture line would be longer and the risk of bleeding would increase. Therefore, we performed hemi-root replacement instead. Although the indications for this technique are limited, it may be an option in some cases of acute aortic dissection with an impaired aortic valve and dissection of the right coronary sinus of Valsalva and the non-coronary sinus of Valsalva.

## CONFLICT OF INTEREST STATEMENT

None declared.

## FUNDING

The authors received no funding in relation to the manuscript.

## Supplementary Material

Supplementary_Video_1_rjac129
